# Immunological Responses to Transgene-Modified Neural Stem Cells After Transplantation

**DOI:** 10.3389/fimmu.2021.697203

**Published:** 2021-06-23

**Authors:** Naili Wei, Zhenxing Sun, Jimei Yu, Yanfei Jia, Peiqi Zheng, Hailiang Tang, Jian Chen

**Affiliations:** ^1^ Department of Neurosurgery, The First Affiliated Hospital of Shantou University Medical College, Guangdong, China; ^2^ Department of Neurosurgery, Beijing Tsinghua Changgung Hospital, School of Clinical Medicine, Tsinghua University, Beijing, China; ^3^ Department of Nursing, Huashan Hospital North, Fudan University, Shanghai, China; ^4^ Department of Neurosurgery, The Second Hospital of Lanzhou University, Lanzhou, China; ^5^ Department of Neurosurgery, Huashan Hospital, Fudan University, Shanghai, China

**Keywords:** neural stem cells, major histocompatibility complex, magnetotactic bacteria, immunological response, transgenic modification, *mms6*

## Abstract

Neural stem cell (NSC) therapy is a promising therapeutic strategy for stroke. Researchers have frequently carried out genetic modification or gene editing of stem cells to improve survival or therapeutic function. However, NSC transplantation carries the risk of immune rejection, and genetic modification or gene-editing might further increase this risk. For instance, recent studies have reported on manipulating the stem cell genome and transplantation *via* the insertion of an exogenous gene derived from magnetotactic bacteria. However, whether transgene-modified stem cells are capable of inducing immunological reactions has not been explored. Although NSCs rarely express the major histocompatibility complex (MHC), they can still cause some immunological issues. To investigate whether transgene-modified NSCs aggravate immunological responses, we detected the changes in peripheral immune organs and intracerebral astrocytes, glial cells, and MHC-I and MHC-II molecules after the injection of GFP-labeled or *mms6*-GFP-labeled NSCs in a rat model. Xenogeneic human embryonic kidney (HEK-293T) cells were grafted as a positive control group. Our results indicated that xenogeneic cell transplantation resulted in a strong peripheral splenic response, increased astrocytes, enhanced microglial responses, and upregulation of MHC-I and MHC-II expression on the third day of transplantation. But they decreased obviously except Iba-1 positive cells and MHC-II expression. When injection of both *mms6*-GFP-labeled NSCs and GFP-labeled NSCs also induced similar responses as HEK-293T cells on the third days, but MHC-I and MHC-II expression decreased 3 weeks after transplantation. In addition, *mms6* transgene-modified NSCs did not produce peripheral splenic response responses as well as astrocytes, microglial cells, MHC-I and MHC-II positive cells responses when compared with non-modified NSCs. The present study provides preliminary evidence that transgenic modification does not aggravate immunological responses in NSC transplantation.

## Introduction

Neural stem cell (NSC) therapy has emerged as a promising treatment strategy for disorders such as degenerative diseases, traumatic brain injury, and ischemic stroke (1, 2). Stem cell therapy has been booming in recent decades (2), but unfortunately, stem cells can no longer satisfy the demands of research or treatment since they cannot be controlled as needed when grafted into hosts (3–5). However, researchers can modify stem cells to meet the research needs *in vivo* by inserting a special gene, protein, or particle (3, 6). Recently, a few studies have reported the manipulation of mesenchymal stem cells (MSCs) through the insertion of material derived exogenously from magnetotactic bacteria (3, 6, 7). Transgene-modified stem cells can produce endogenous magnetic nanoparticles, which MRI can be tracked *in vivo* (3, 6). These reports are exciting and promising; however, transgene-modified stem cells also pose uncertain risks to immunological security. As we are only in the early stages of allogeneic stem cell modification, few studies to date have focused on the possible immunological responses following the transplantation of these modified stem cells.

Class I and class II major histocompatibility complex (MHC) molecules cooperate and co-regulate the immunological response process (8–11). They play a key role in the induction of xenograft rejection, although only a small proportion express and possess exogenous immunogenicity (8, 12–15). In a previous study, NSCs were transplanted from transgenic pigs into the striatum of rats; these xenogeneic NSCs survived for a long time and stably expressed exogenous genes (15). NSCs express a low level of MHC antigen and reduce the sensitivity of cytotoxic T lymphocytes to dissolution following NSC transplantation (16–18). Using *in vitro* models, several studies have demonstrated that neither allogeneic NSCs (19–21) nor progenitor cells (12, 17) induce a severe immunological response. Previous studies have also reported that allogeneic MSCs activate striking immunological responses (22, 23); however, the responses prompted by transgene-modified NSCs have yet to be studied. Therefore, it is necessary to investigate the immunological responses of transgene-modified NSCs ahead of clinical studies.

NSCs were transplanted after modification with the magnetosome membrane-specific gene (*mms6*) derived from magnetotactic bacteria (24). The *mms6* gene does not exist in mammalian genomes; instead, it is native to magnetotactic bacteria and is responsible for the bioassimilated synthesis of endogenous intracytoplasmic magnetic nanoparticles that can be detected by MRI (3, 6). This property has seen the *mms6* gene employed for stem cell modification, as it allows the fate of stem cells to be tracked; thus, its use may be a promising stem cell modification strategy for *in vivo* tracking. Furthermore, the *mms6* gene may also have different operation mechanisms in mammalian cells, and it has demonstrated no deleterious effect on stem cell proliferation, migration, or differentiation. However, no related immunological studies have been conducted to date.

In the present study, the immunological responses induced by transgene-modified NSCs analyzed astrocytes, microglia, and MHC-I and MHC-II molecules at 3 days and 3 weeks after transplantation, respectively. At the same time, the responses of the spleen and kidney were also detected.

## Materials and Methods

### Materials

Dulbecco’s Modified Eagle Medium (DMEM)/Nutrient Mixture F-12 (F12) and fetal bovine serum (FBS) were bought from Gibco. B27 supplement (17504044), N2 supplement (17502048), and trypsin were obtained from Thermo Fisher. Anti-GFP (ab290), anti-GFAP (ab7260, Abcam), anti-IBA-1 (ab178847, Abcam), anti-MHC-I (ab134189, Abcam), anti-MHC-II (ab23990, Abcam), and anti-nestin (ab11306, Abcam) were procured from Abcam. Neonatal Wistar rats and 12-week-old Wistar rats supplied by the Shanghai Laboratory Animal Center (Shanghai, China) were used in our experiments.

The study protocol was approved by the Institutional Animal Care and Use Committee of Shantou University. All procedures were conducted in adherence with the Guidelines for Animal Experimentation of Shantou University and the Chinese Guidelines for the Care and Use of Laboratory Animals.

### Isolation and Culture of NSCs

NSCs were isolated and cultured as described previously (15). In brief, the forebrains of the neonatal Wistar rats were dissected within 24 hours of birth to isolate the NSCs. Subsequently, the NSCs were grown as free-floating neurospheres and cultured in DMEM/F12 (1:1) medium containing 2% B27 and b-FGF (20 μg. L^-1^), 5% N2, and 10% double antibodies (penicillin and streptomycin). The culture medium was replaced every 7 days.

### The Expression of *mms6* in NSCs

After codon optimization for mammalian expression, the AMB-1 *mms6* deoxyribonucelic acid (DNA) bacterial sequence of *Magnetospirillum magneticum* was cloned and synthesized into a lentivirus vector (pHBLV-CMV-MCS-3FLAG-EF1-ZsGreen-T2A-PURO). Additionally, a control vector without *mms6* expression was also synthesized. Successful expression was verified by polymerase chain reaction (PCR) and gene sequencing. The *mms6* primers used for PCR amplification were: *mms6*-F, GGATCTATTTCCGGTGAATTCGCCACCATGGGATCCGCCACCATGCC; and *mms6*-R, TAAGC-TTGGT ACCGAGGATCC AGCCAGAGCGTCCCTAAGTT.

Before lentiviral transfection, NSCs were cultured into single cells on 6-well plates pre-coated with Matrigel. Lentiviral transfection was carried out after the NSCs had spread in the single cells and 70% confluence had been reached. Then, 45 μl lentivirus (10^9^/ml) was added to each well (multiplicity of infection [MOI] approx. 10:1). After overnight incubation, the medium was replaced. After 3 days, untransfected cells (2.5 μg/ml) were screened through the addition of puromycin antibiotics, and the medium was replaced 3 days later. Photographs were taken with a fluorescence microscope, and the transfection efficiency was determined.

Human embryonic kidney cells (293T), which were cultured in DMEM supplemented with 10% FBS, were used as the positive control for immunological experiments. The lentiviral vector was added to each well to label the 293T cells with GFP (MOI approx. 1:1).

### Transplantation Procedures

According to the grouping method, 32 rats were randomly divided into the control group (n=8), 293T group (n=8), GFP-NSC group (n=8), and *mms6*-GFP-NSC group (n=8). Subsequently, GFP-labeled 293T cells, GFP-labeled NSCs, and *mms6*-GFP-labeled NSCs were injected into the right striatum of the rats in the corresponding groups. NSCs and 293T cells were processed into single cells, and the cell concentration was adjusted to 1 × 10^8^/ml. The cells (5 × 10^5^ cells, 5 μl) were transplanted into the right striatum of the rats (AP, 0.0 mm; ML, 2.0 mm; and DV, 3.5 mm). The control rats were injected with an equivalent amount of phosphate-buffered saline (PBS). To prevent postoperative infection, intraperitoneal penicillin injections were given at intervals of 3 days.

### Bodyweight Changes and Immune Organ Responses

Each rat’s body weight (BW) was measured before transplantation and on days 3, 7, 14, and 21 following transplantation. On day 3 after transplantation, 3 rats from each group were sacrificed; on day 21 post-transplantation, the other 5 rats were sacrificed. Subsequently, the weights of the brain, spleen and right kidney were measured. All rats were sacrificed through intracardiac perfusion with 4% paraformaldehyde under anesthesia.

### Histological Process

Frozen brain, spleen, and kidney tissue samples were sliced using a freezing microtome (Leica). The brain sections (25 μm in thickness) were subjected to immunohistochemical (IHC), immunofluorescence (IF), and Prussian blue staining, while the spleen and kidney sections (25 μm in thickness) were subjected to hematoxylin and eosin (HE) staining.

IHC staining was performed in line with previously published protocols (25). Briefly, primary antibodies against MHC-I (ab134189, Abcam, 1:100) and MHC-II (ab23990, Abcam, 1:100), and the secondary antibodies anti-rabbit IgG (ab205718, Abcam, 1:2000) and anti-mouse IgG (ab205719, Abcam, 1:2000) were used to detect the immunological responses to cell transplantation. After 24-hour incubation at 4°C followed by washing, the Histostain TM-SP kit was used to detect and visualize signals, in line with the manufacturer’s protocol.

IF staining was performed according to previously published protocols (26). Briefly, paraffin brain sections were deparaffinized and rehydrated before immunostaining, which was followed by overnight incubation in blocking solution containing primary antibodies (anti-GFAP, Abcam, ab7260 1:100; anti-IBA-1, Abcam, ab178847, Abcam, 1:100; anti-Nestin, Abcam, ab221660) at room temperature. After washing with PBS, the sections were further incubated with the corresponding secondary antibodies (goat polyclonal secondary antibody to Rabbit, 150079 1:500) for 2 hours at room temperature. Then, the sections were washed and loaded onto a coverslip after adding mounting medium to each section. A confocal laser scanning microscope (Nikon, Japan) was used to scan the samples. Slices from the injection site of cell transplantation were used for staining. Positive markers in 4 slices from 3 rats in each group were counted for analysis. A Nikon Element reviewer (Nikon, Japan) was used for image analysis.

### Cell Proliferation Experiment

Two types of NSCs (*mms6*-GFP-NSCs and GFP-NSCs) were cultured under the same conditions: 5% CO_2_ and 20% O_2_. To determine the effects of different conditions on cell proliferation, the Cell Counting Kit-8 (CCK-8) assay was used to detect the OD450 value of each well using an enzyme labeling instrument. Two groups of cells were plated in a 96-well plate pre-coated with Matrigel (2 × 10^3^ cells/hole, 200 μl serum-free NSC culture medium/hole). At 12 hours and 1, 2, 4, and 8 days, photographs of the cells in each group were taken.

### Statistical Analysis

Data were expressed as mean ± standard error of the mean. SPSS 12.0 was employed for statistical analysis of all experimental data; a *p*-value <0.05 was taken to indicate statistical significance. The nonparametric rank-sum test and two-sample independent t-test were used to compare the differences between the different groups.

## Results

### Immune Organ Responses at the Early and Late Stages of NSC Transplantation

Primary NSCs were transfected with lentivirus (**Supplemental Figure S1A**), and both *mms6*-GFP-NSCs and GFP-NSCs were screened with puromycin and expressed GFP fluorescence (**Supplemental Figures S1C, D**). The results of PCR showed that *mms6*-GFP NSCs had a positive band of around 100 kb, whereas GFP NSCs did not have such band (**Supplemental Figure S1B**), indicating that *mms6* had been expressed in the former successfully. After cell transplantation, nestin + GFP-labeled cells were considered to be exogenous NSCs (**Supplemental Figures S1E, F**). To better understand immune rejection by the central nervous system after allogeneic cell transplantation, we used the HEK-293t cell transplantation and normal saline transplantation groups as positive and negative control groups, respectively. To investigate whether transplantation of transgenic *mms6*-modified NSCs elicits immune organ responses, the structures and cell morphology of the brain, spleen, and kidney tissues of rats were assessed on days 3 and 21 after cell transplantation. The changes in BW were also recorded.

**Figure 1:**
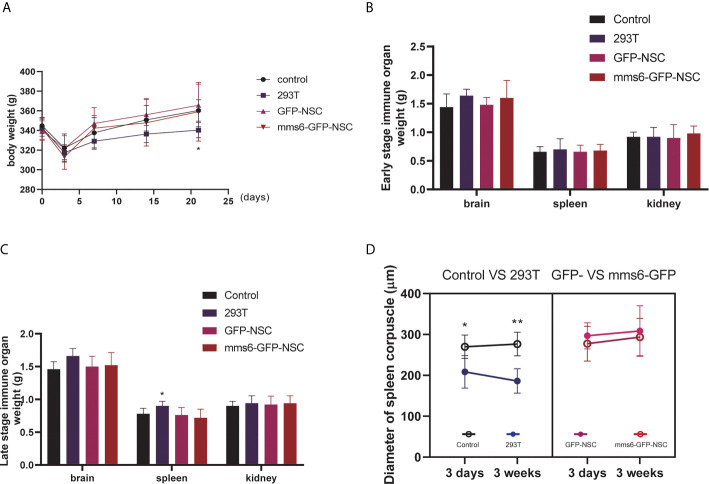
Periphery immune organ responses. **(A)** Body weight changes in rats of control group, 293T group, GFP-NSCs group, and *mms6*-GFP-NSCs after cell transplantation from pre-operation to 21 days post-operation. **(B)** Comparisons of weights among four groups at the early stage of transplantation. **(C)** Comparisons of weights among four groups at the later stage of transplantation. **(D)** Diameters of spleen corpuscle in rats of four groups at the early and later stages of transplantation. At the early and later stages, the diameters of spleen corpuscle gradually decreased compared with those of control group. However, *mms6*-GFP-NSCs transplantation did not induce any splenic changes in the corpuscle. (*control group *vs.* 293T *p*< 0.05; **control group *vs.* 293T *p*< 0.01).

**Figure 2:**
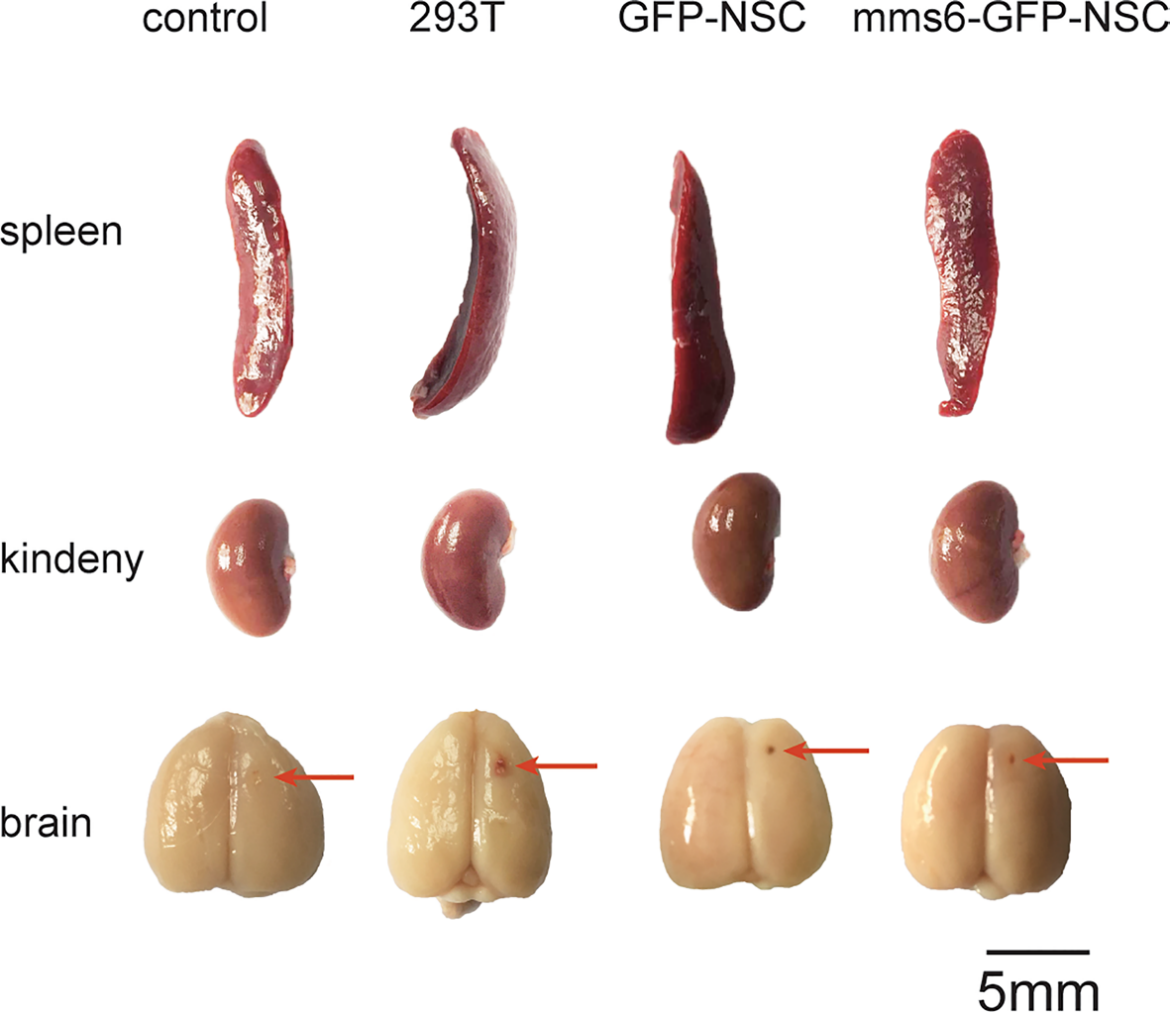
Morphological changes in the brain, spleen, and kidney of rats among the four groups on the 21st day following transplantation. No obvious difference in the morphology of these organs could be seen among groups. The red arrows indicate the needle path in the NSCs transplantation area.

After cell transplantation, rats in all the groups showed decreased BW compared to the pretransplantation level. Three days later, their BW had gradually recovered; however, the rats given a 293T cell injection had a lower BW than the controls (**Figure 1A**). No significant difference in BW change was detected between the *mms6*-GFP-NSC and GFP-NSC groups after transplantation. On day 3, after cell transplantation, the morphology and weights of the brain, spleen and kidney showed no obvious changes (**Figure 1B**). However, in the 293T group, the spleen weight increased compared to that in the control group (0.90 ± 0.071 g *vs.* 0.78 ± 0.084 g, *p*=0.04) (**Figure 1C**), but no significant difference was seen in the brain or kidney weight at the later stage of cell transplantation. A blood inflammatory reaction was observed in the transplantation area on the brain surface of rats in the 293T group (**Figure 2**).

As the largest peripheral immune organ, the spleen plays an important role in the human body’s immune response. During immunological rejection of allogeneic grafts, the spleen may become progressively enlarged. According to our results, the intracerebral injection of xenogeneic cells elicited immune organ responses; however, the transgenic *mms6*-modified NSCs did not elicit peripheral immune organ responses.

**Figure 3:**
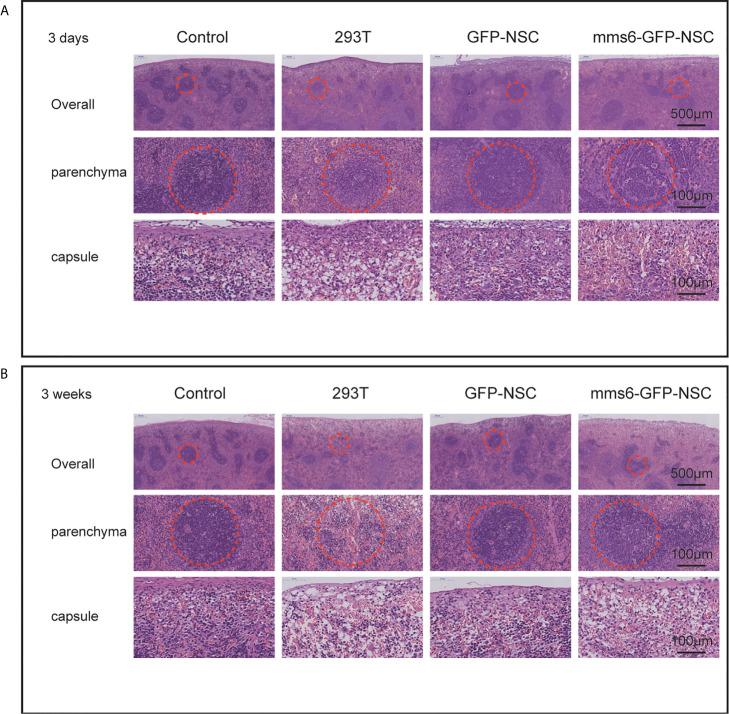
Hematoxylin and eosin staining of the spleen tissues. **(A)** Morphology of spleen tissues in rats among four groups on the third day of transplantation. ‘Overall’ displays the organizational structure and morphology of the spleen including capsule and parenchyma. ‘Parenchyma’ shows the splenic corpuscleogenesis centers of rats among the four groups. Structural destruction was seen in the splenic corpuscles of 293T group. **(B)** Morphology of spleen tissues in rats among the four groups on the 21st day of transplantation. Most splenic corpuscleogenesis centers of parenchyma in 293T group disappeared. The dotted circle indicates splenic corpuscleogenesis centers.

The organizational structures and cell morphologies of the brain, spleen, and kidneys of the rats were also examined by HE staining. Specifically, the diameters of the splenic and renal corpuscles were the focus of HE staining. Our results demonstrated that only the spleens of the 293T group exhibited progressive immunological responses from the early stage to the late stage of cell transplantation. In comparison, the kidneys did not exhibit an obvious response to cell transplantation at either stage in any of the groups. On day 3 after cell transplantation, the spleen capsule in the 293T group became edematous, and a normal boundary could not be identified in the parenchyma. Also, the structure of the splenic corpuscleogenesis center was destroyed by local exudation (**Figure 3A**).

Additionally, the diameter of the splenic corpuscle in the 293T group was smaller than that in the control group (**Figure 1D**). On day 21 after transplantation, the normal structure of the splenic corpuscleogenesis disappeared (**Figure 3B**). Moreover, the mean diameter of the splenic corpuscle of the rats that received 293T transplantation was significantly decreased compared to that of the control group (186.24 ± 14.95 μm *vs.* 276.62 ± 14.38 μm, *p*=0.005). However, the transplantation of *mms6*-modified NSCs did not elicit splenic responses compared to the control group (308.38 ± 30.95 μm *vs.* 276.62 ± 14.38 μm, *p*=0.552) or GFP-NSC group (308.38 ± 30.95 μm *vs.* 293.63 ± 22.82 μm, *p*=0.716).

**Figure 4:**
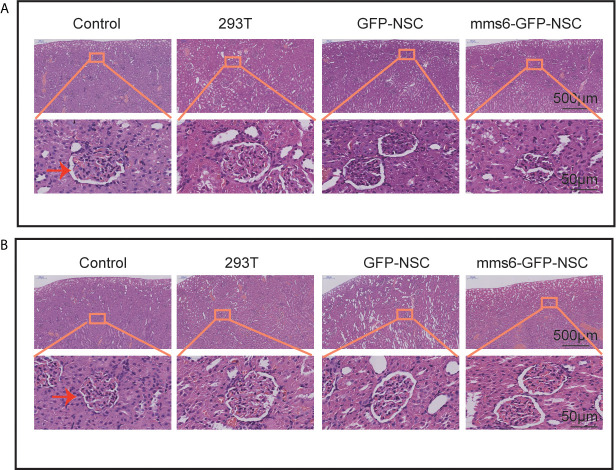
Hematoxylin and eosin staining of kidney tissues. No obvious morphological changes were seen in the capsule, parenchyma, and glomerulus of rats among four groups. **(A)** Morphology of kidney tissues in rats among four groups on the third day of transplantation. **(B)** Morphology of kidney tissues in rats among four groups on the third week of transplantation. The orange rectangle indicates renal corpuscle.

As observed in the representative HE staining images in **Figure 4**, no significant differences in kidney structure or cell morphology were observed between the four groups at the early or late stage of cell transplantation.

### Cell Transplantation Elicits Astrocyte and Microglial Cell Activation

**Figure 5:**
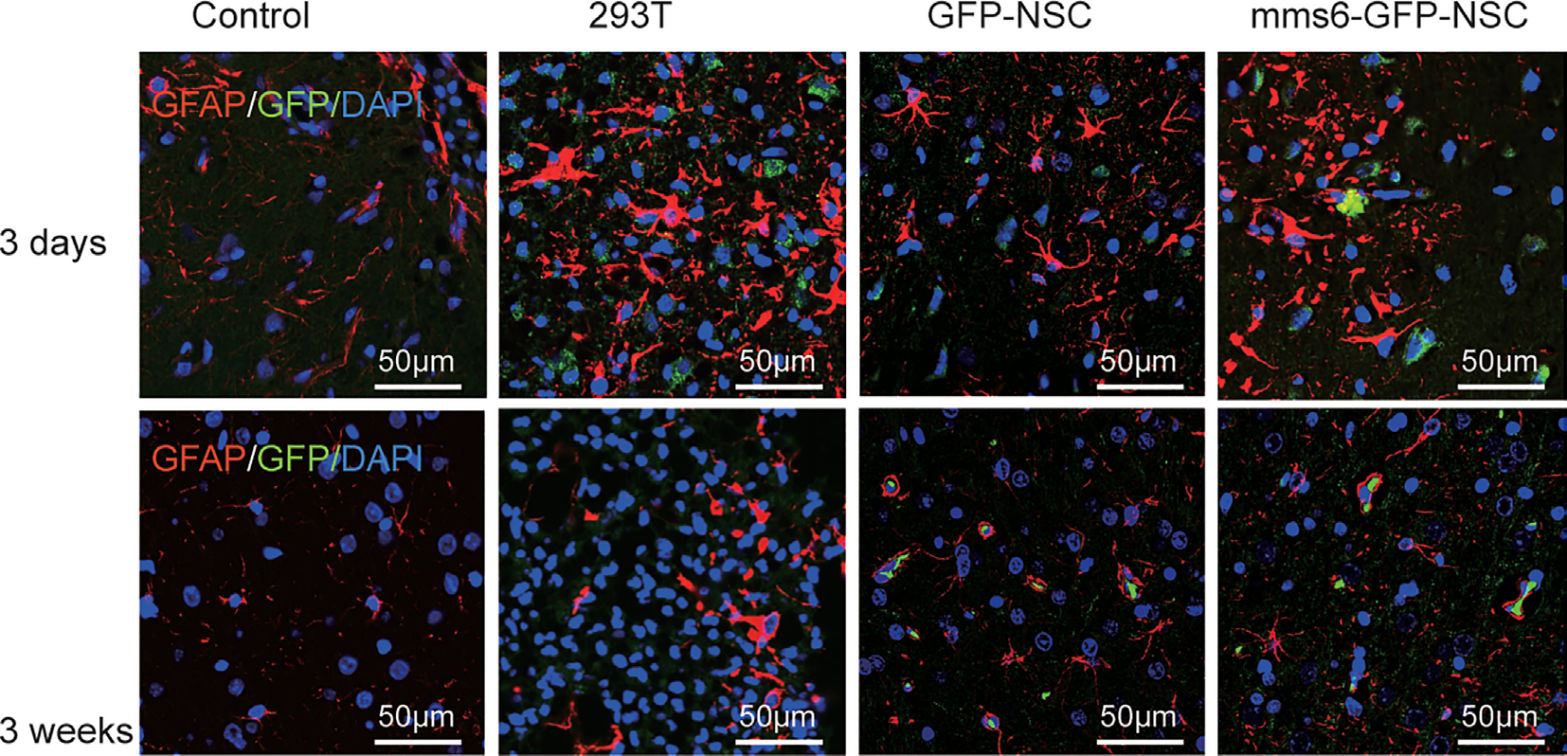
The glial cells (staining marker GFAP: red) were accumulated around and immersed at the site of transplantation in all the groups at the early stage of transplantation (upper panel); however, they decreased significantly at the later stage (lower panel).

To investigate the intracerebral immunological responses to the transplantation of transgenic *mms6*-modified NSCs, astrocytes and microglial cells in the transplantation area were detected by IF staining using GFAP and Iba-1. NSCs and 293T cells were labeled with GFP. As a biomarker of brain injury, GFAP represents the degree of inflammatory injury at the transplantation site (25, 27). As shown in **Figures 5** and **9A**, GFAP-positive cells were accumulated around and immersed at the transplantation site in all groups of rats on day 3 after transplantation; however, they had decreased significantly on day 21 after transplantation. Compared with the other groups, the group with allogeneic 293T cell transplantation exhibited more intensive astrocyte cell responses even at a later stage. NSC transplantation did not induce GFAP-positive cell responses compared to the control group at either stage (early stage: *mms6*-GFP-NSCs *vs.* Control, F=0.475, *p*=0.341; GFP-NSCs *vs.* Control, F=0.044, *p*=0.502; later stage *mms6*-GFP-NSCs *vs.* control, F=0.044, *p*=0.502; GFP-NSCs *vs.* Control, F=1.559, *p*=0.480). These data indicate that the inflammatory status in 293T cell-treated rats was not induced by brain injury induced by transplantation. Furthermore, these data also show that the group with transgene-modified NSCs did not show aggravated inflammation compared to the GFP-NSC group.

**Figure 6:**
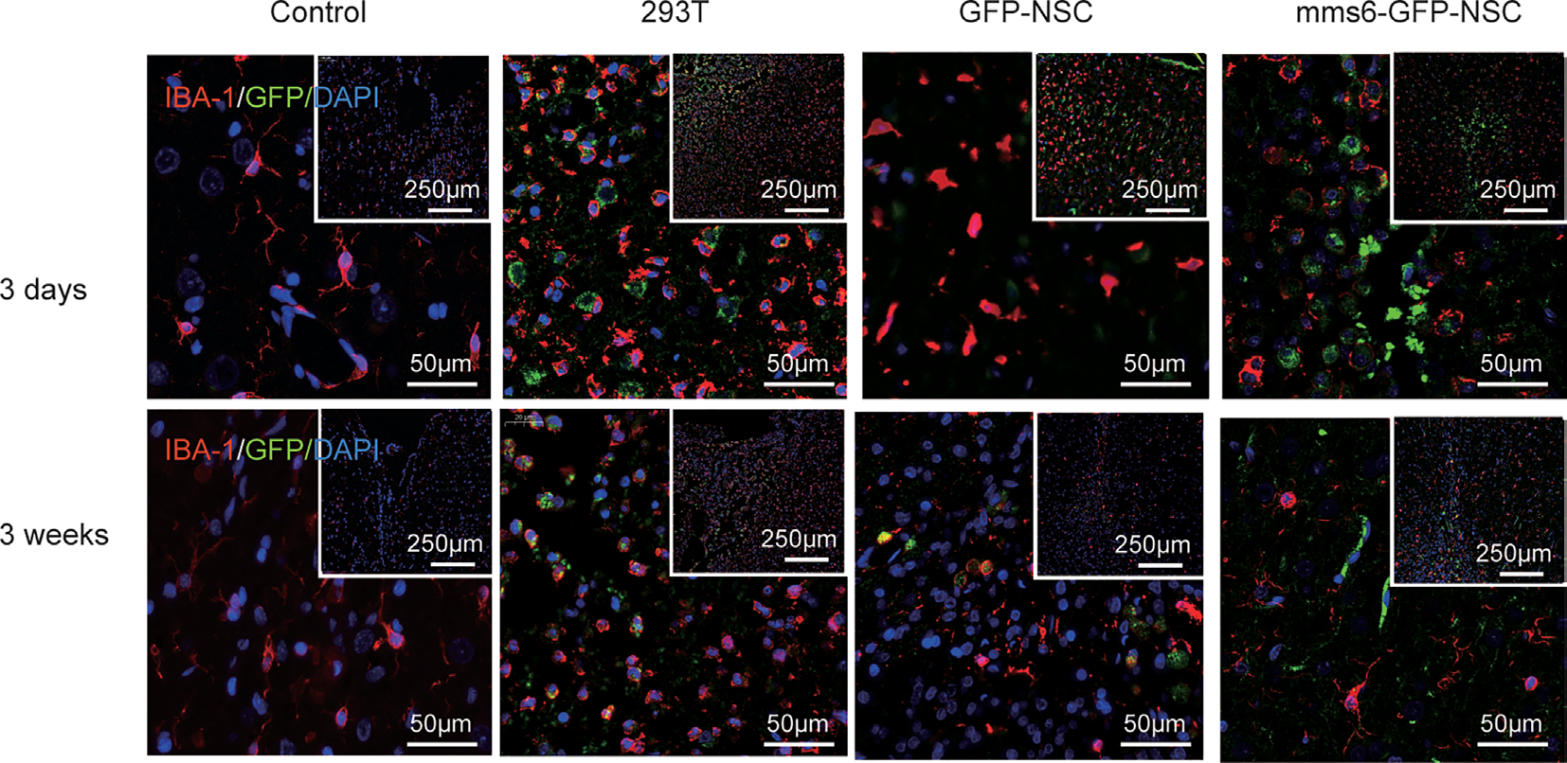
Xenogenic cells transplantation elicited obvious microglial cells (staining marker: Iba-1, red) responses, especially at later stage following transplantation (lower panel). NSCs transplantation elicited fewer microglial cells at early stage (upper panel). However, transgene *mms6*-modification did not aggravate microglial cell responses.

Microglial cells in the brain are recognized as resident macrophages, which play a critical role in regulating inflammatory responses and immunological reactions (23, 25). In the present study, Iba-1 was utilized as a marker for microglial cells since it is also regarded as analogous to proteins characterized by xenograft inflammation factor 1 (AIF-1). As exhibited in **Figures 6** and **9B**, both *mms6*-modified NSCs and GFP-NSCs elicited Iba-1 cell responses compared to the control group at an early stage (*mms6*-GFP-NSCs *vs.* Control, F=2.025, *p* < 0.000; GFP-NSCs *vs.* Control, F=0.12, *p* < 0.000) but not at later stages (*mms6*-GFP-NSCs *vs.* Control, F=4.587, *p*=0.118; GFP-NSCs *vs.* Control, F=3.556, *p*=0.073). However, the reactions differed from those in the 293T group. On day 3 after transplantation, Iba-1 positive cells of rats in the control group, GFP-NSC, and *mms6*-GFP-NSC groups were immersed in the transplantation site; however, they had decreased significantly on day 21 after transplantation.

In contrast, Iba-1 positive cells in the transplantation area in the 293T rats were significantly increased at the later stages, and the GFP-positive cells had even disappeared. These results indicate that 293T cell transplantation promoted the elimination of xenograft cells by microglial cells. The state of microglial cells also represents an immunological rejection process.

### MHC Regulates Immunological Responses in Xenogeneic Cells but Not in Transgene-Modified NSCs

**Figure 7:**
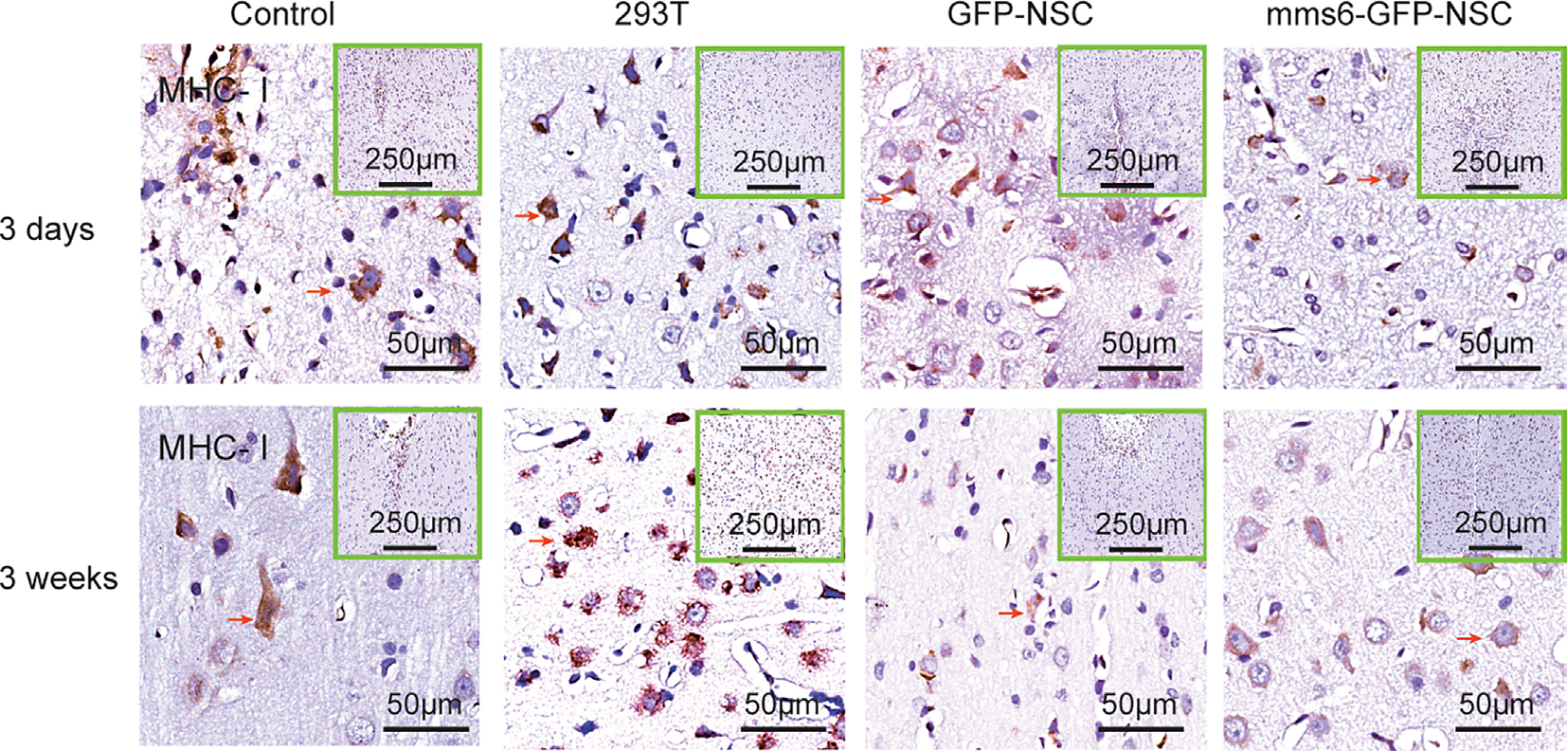
Immunohistochemistry of major histocompatibility complex (MHC)-I molecule expression in the transplantation areas of brains among the four groups on the third day (upper panel) and 21^st^ day (lower panel) post-operation. The red arrows indicate typical example of positive cells in each group.

Immunological rejection has been widely accepted as being dominated by MHC molecules, including MHC I and MHC-II (8, 9). MHC-II is scarcely expressed in the brain, whereas MHC I is mainly expressed in reactive microglial cells (15, 16, 28). As illustrated in **Figures 7** and **9C**, MHC I-positive cells appeared to be activated by brain injury in each group. Like the changes in astrocytes, in all groups, MHC I-positive cells also accumulated around the transplantation sites on day 3 after transplantation but had decreased significantly by day 21 after transplantation. At the later stage, MHC I-positive cells were activated more intensively in the 293T group than in the other three groups. Furthermore, transgenic *mms6*-modified NSCs did not elicit greater MHC I responses than did GFP-NSCs (*mms6*-GFP-NSCs *vs.* GFP-NSCs, F=0.108, *p*=0.075), although they appeared to elicit MHC I responses when compared to the control group at the early (*mms6*-GFP-NSCs *vs.* control, F=2.998, *p*=0.046) and later stages (*mms6*-GFP-NSCs *vs.* Control, F=3.515, *p*=0.004) after transplantation.

**Figure 8:**
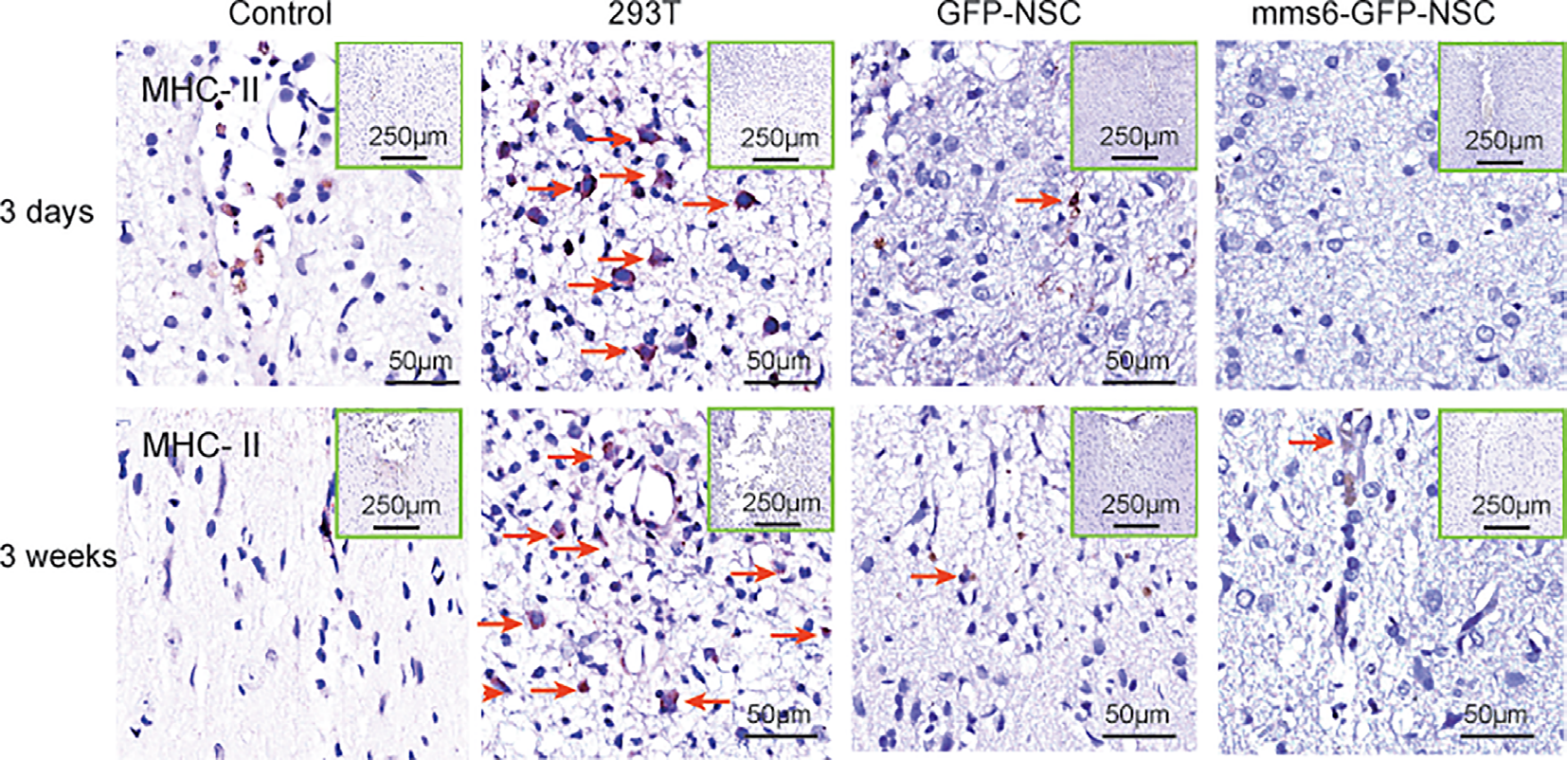
Immunohistochemistry of major histocompatibility complex (MHC)-II molecule expression in the transplantation areas of the brain among the four groups on the third day (upper panel) and 21^st^ day (lower panel) post-operation. The red arrows indicate typical example of positive cells in each group.

**Figure 9:**
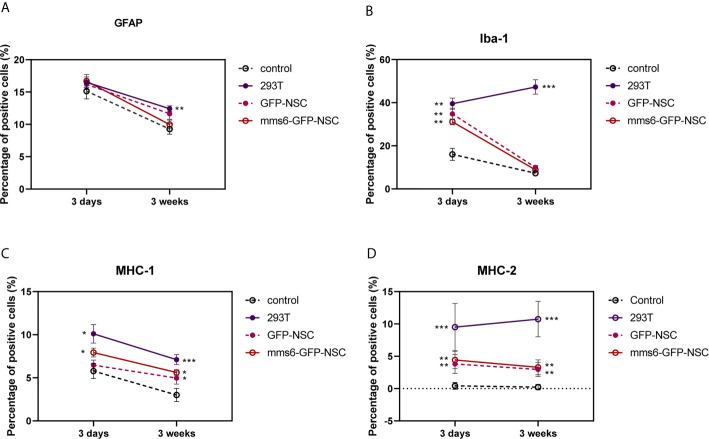
Quantitative analysis of GFAP **(A)**, Iba-1 **(B)**, major histocompatibility complex (MHC)-I molecule **(C)**, and MHC-II **(D)** molecule expression in transplantation areas of brains among four groups on the third day and 21^st^ day post-operation. **(A)** Transplantation operation recruited 293T GFAP+ cells in the operation areas among all groups at the early stage, but the cell number decreased at the later stage. Obviously, the GFAP response was still seen in 293T group compared with control group. **(B)** At the early stage of transplantation, xenogeneic 293T cell transplantation induced obvious Iba-1+ expression compared with that in control group, and the stronger status was observed at the later stage. In contrast, the exogenous gene *mms6*-modified NSCs transplantation did not induce obvious Iba-1 expression at either the early or the late stage compared with that in GFP-NSCs group. **(C)** Transplantation operation induced MHC-I expression at the early stage among all groups. Xenogeneic 293T cell transplantation induced more obvious MHC-I expression compared with that in control group, and the stronger status was observed at the later stage. In comparison, the exogenous gene *mms6*-modified NSCs transplantation did not induce obvious MHC-I expression at either the early or later stage relative to that in GFP-NSCs group. **(D)** Almost no expression of MHC-II molecule was detected in the transplantation areas of rats among all groups except for 293T group. (*control group *vs.* 293T, p< 0.05; **control group *vs.* 293T, *p*< 0.01; ***control group *vs.* 293T, *p<* 0.001).

MHC-II-positive cells were not detected in tissue samples from the control group. However, they were found in the transplantation area of the 293T, *mms6*-NSC, and GFP-NSC groups (**Figure 8**). As demonstrated by previous studies (15, 16, 28), NSC transplantation can elicit a few MHC-II positive cell responses. However, *mms6*-modification did not aggravate the response (*mms6*-GFP-NSCs *vs.* GFP-NSCs, F=0.008, *p*=0.465). The MHC-II-positive cell responses were found to be similar to the changes observed in Iba-1-positive cells (**Figures 8** and **9D**). In the 293T group, the number of MHC-II-positive cells progressively increased during the cell transplantation process. In contrast, MHC-II-positive cells were rarely present in the transplantation area of rats in the GFP-NSC and *mms6*-GFP-NSC groups; however, their numbers were still higher than those in the control group. There was no significant difference in MHC-II-positive cells between the two groups on days 3 and 21 after transplantation.

### The Expression of mms6 Does Not Affect NSC Proliferation

Previous studies have confirmed that the expression of magnetotactic bacterial genes does not cause changes in cell proliferation or differentiation (3). Our study used the lentiviral method to stably express both the *mms6* and *GFP* genes in NSCs, while the control cells only expressed the *GFP* gene. After 8 days of culture, there was no significant difference in the proliferation rate of NSCs between the two groups, as evidenced by the CCK-8 experimental results (**Supplemental Figure S2**). This finding is consistent with the results of a previous study (3).

## Discussion

The present study aimed to investigate peripheral and intracerebral immune responses following the transplantation of transgenic *mms6*-modified NSCs. Our preliminary observations of peripheral immune organ responses demonstrate that exogenous gene-modified NSCs did not elicit splenic responses similar to those in xenograft cells. The intracerebral inflammatory and immunological responses were also studied. We found that the transplantation of transgene-modified NSCs increased astrocyte and microglial cell responses and enhanced the MHC-I and MHC-II-positive cell responses. However, there was no evidence to support the idea that exogenous gene-modified NSCs aggravated immune responses compared with GFP-NSCs.

Astrocytes and microglial cells are critical executors in inflammatory and immunological responses (25–27, 29, 30). Astrocytes are responsible for repair following injury or damage and induce the elevated production of inflammatory factors, further activating these cells to migrate toward the injured sites (26, 27, 30). Our results indicated that the responses of astrocytes to NSC transplantation were derived from the transplantation procedure since similar changes were also observed at the transplantation site of rats in the control group given PBS injection alone. GFAP, a biomarker for astrocytes, also serves as a biomarker of brain damage, with more severe brain damage being linked with a higher level of GFAP accumulation in focal lesions. At the later stage after transplantation, the number of GFAP-positive cells at the transplantation site was decreased in the *mms6*-GFP-NSC and GFP-NSC groups, which was similar to what was observed in the control group.

In contrast, Iba-1-positive microglial cells are responsible for the elimination of severely damaged or dead cells and for phagocytizing cell debris and xenogenic cells (23, 31–33). When exogenous cells are injected into the brain, many quiescent microglial cells can be reactivated (23, 34, 35), but they only phagocytize the identified heterogeneous cells. Our results also support this hypothesis. After the elimination of 293T cells, the number of microglial cells was observed to increase gradually. Other causes might also lead to decreases in GFP-NSCs and *mms6*-GFP-NSCs at a later stage.

In the present study, MHC molecules were also detected. When exogenous cells are injected into the brain, they are identified as xenogenic cells due to their MHC expression, and they are phagocytized (36, 37). Our results indicated that the number of MHC-I-positive cells increased on day 3 but decreased on day 21 after transplantation, which was similar to the changes observed in astrocytes. Meanwhile, the changes in MHC-II-positive cells were similar to those in Iba-1-positive cells. However, these two molecules are thought to be expressed in microglial cells rather than astrocytes in the brain (34). It is speculated that both MHC-I-positive cells and MHC-II molecules are activated by exogenous cell transplantation; however, they play different roles in the immunological response to NSC transplantation. The former is regulated by leukemia inhibitory factor (LIF), while the latter is activated by IFN-γ and TNF-β (10, 38). LIF is a critical signal of brain injury and activates microglial cells to repair and induce neurogenesis (39, 40). Both interferon-gamma (IFN-γ) and tumor necrosis factor (TNF)-β are important cytotoxic factors that can promote brain microglial cells and macrophages to eliminate xenogenic cells (11, 41, 42). MHC-I has been reported to assist in immune recognition in reconstructing synapse connections (43, 44), whereas MHC-II helps to clear mismatched xenogenic cells (38, 45). Therefore, the responses of MHC-II-positive microglial cells at later stages better reflect the occurrence of heterologous cell rejection. Considering this, our data support the notion that *mms6*-modified NSCs did not result in immunological rejection.

We have studied the immune rejection of allogeneic gene-modified stem cell transplantation, but we have not systematically evaluated the immune response at the levels of cytokines and inflammatory factors. Several studies have proved that microglia activation can result in antigen presentation and the secretion of cytokines such as interleukin-1 beta (IL-1β), IFN-γ, and TNF-α (46–50). However, one study found that IFN-γ treatment induced a degree of activation of MHC-II but not MHC-II (46), while another reported that TNF-α did not impact the numbers of MHC-II immunoreactive cells (51). In contrast, the cytokine IL-1β has been shown to inhibit MHC-II expression (52). Therefore, IL-1β, IFN-γ, and TNF-α participate in the regulation of MHC-I activation, but only IL-1β is involved in the activation of MHC-II (53, 54). In our study, grafting of HEK-293T cells induced a strong immunological reaction. Iba-1 positive cells and MHC-II positive cells exhibited similar changes both on day 3 and day 21 after transplantation. MHC-I-positive cells exhibit changes in the brain similar to those seen with brain injury (50). Considering the above evidence, we speculated that the levels of cytokines and inflammatory factors increased sharply in the early stage of transplantation but decreased as the brain injury recovered. At the later stage of transplantation, some cytokines involved in class II MHC activation, such as IL-1β, still play an important role in regulating immunological reactions. We will investigate the changes in cytokines and inflammatory factors in future studies to fully evaluate the immunological responses to allogeneic and transgenic modification.

The gene *mms6 *originates from magnetosome membrane-specific gene clusters of the *Magnetospirillum magneticum* strain AMB-1 (24). It encodes a critical small molecular protein, MMS6, consisting of 60 amino acids, and promotes the formation of uniform magnetic nanocrystals *in vitro* (24). Recently, it has been reported that mms6 can be inserted into a human or mammalian genome for manipulation (3, 6). MSCs expressing mms6 can produce intracellular magnetic particles (3). This technology can overcome the problems of stem-cell tracking *in vivo*, which also marks the beginning of the manipulation of exogenous genes from other species. Although no evidence of immunological rejection was found in the present study, further experiments are required. MMS6 is a membrane protein that, theoretically, cannot be transported out of NSCs, and it is recognized as a foreign particle without the help of a special transmembrane transporter. However, whether or not it can be identified as a foreign particle by lysosomes has not been explored, so further studies are warranted. Comparatively speaking, 293T cells express exogenous molecules on the membrane and can be easily identified as xenogenic cells. Other exogenous gene-modified NSCs may produce immunological responses when they encode membrane proteins. However, the present study did not examine the differences in the levels of inflammatory cytokines among the groups. We believe that supplementary research is necessary. In any case, our study provides preliminary evidence that the expression of mms6 does not aggravate the immune response of the central nervous system.

## Conclusion

In conclusion, the present study’s findings demonstrate that xenogeneic cell transplantation induces strong immunological and peripheral immune organ responses; however, exogenous mms6-modified NSC transplantation does not aggravate immunological responses. As gene coding and gene modification technology become increasingly developed, more attention should be paid to safety evaluation, especially the risk of immunological rejection. Our results provide preliminary evidence for the safety evaluation of the preclinical application of a magnetotactic bacteria gene, *mms6*, but it does not mean that its clinical application can be implemented. The clinical application of heterologous genes calls for a more comprehensive safety assessment.

## Data Availability Statement

The original contributions presented in the study are included in the article/**Supplementary Material**. Further inquiries can be directed to the corresponding authors.

## Ethics Statement

The animal study was reviewed and approved by Animal Experimentation of Shantou University. Written informed consent was obtained from the owners for the participation of their animals in this study.

## Author Contributions

JC: conception, supervision, and design of this article. NW, XS, and JY: experiments, data analysis and editing the manuscript. YJ and PZ: Histological procedures data analysis. HT: manuscript editing and data analysis. All authors contributed to the article and approved the submitted version.

## Funding

This work was supported by grants from the National Nature Science Foundation (82001447), China Postdoctoral Science Foundation (2020M682833), Dengfeng Project for the construction of high-level hospitals in Guangdong Province, the First Affiliated Hospital of Shantou University Medical College Supporting Funding (202003-29), 2020 Li Ka Shing Foundation Cross-Disciplinary Research Grant (2020LKSFG11C), and Beijing Tsinghua Changgung Hospital Fund (12015C1045).

## Conflict of Interest

The authors declare that the research was conducted in the absence of any commercial or financial relationships that could be construed as a potential conflict of interest.

## Supplementary Material

Supplementary Figure 1 Verification of xenogenous-gene-modification expression. **(A)** In the primary culture of neural stem cells, spheroids of NSCs could be seen; **(B)** PCR test verified the *mms6* expression in *mms6*-GFP-NSCs. **(C)** GFP-NSCs; M:*mms6*-GFP-NSCs; **(C)** GFP expression in transplanted GFP-NSCs; **(D)** GFP expression in transplanted *mms6*-GFP-NSCs. **(E)** Nestin+ GFP protein was found in the transplantation area of GFP-NSCs group; **(F)** Nestin+ GFP protein was found in the transplantation area of *mms6*-GFP-NSCs group.

Supplementary Figure 2 Expression of *mms6* did not affect NSCs proliferation.
