# Supplementary figures and images for: Immunological Responses to Transgene-Modified Neural Stem Cells After Transplantation
Source: Front Immunol. 2021 Jun 23;12:697203. doi: 10.3389/fimmu.2021.697203 (PMC8262771; doi:10.3389/fimmu.2021.697203)

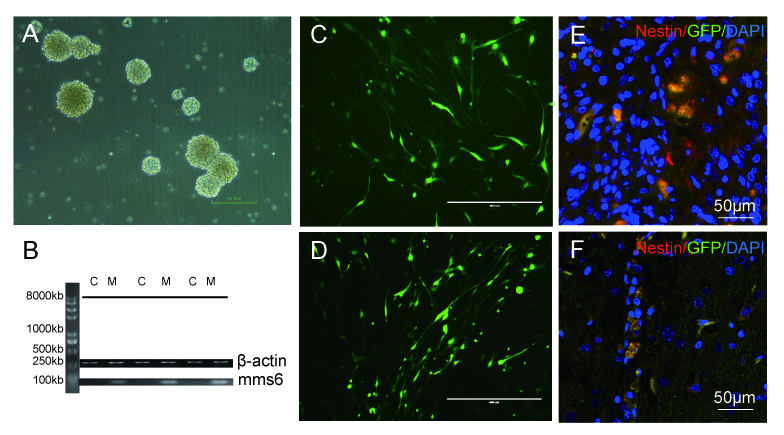

Supplement: Supplementary Figure 1 — Verification of xenogenous-gene-modification expression. (A) In the primary culture of neural stem cells, spheroids of NSCs could be seen; (B) PCR test verified the mms6 expression in mms6-GFP-NSCs. (C) GFP-NSCs; M:mms6-GFP-NSCs; (C) GFP expression in transplanted GFP-NSCs; (D) GFP expression in transplanted mms6-GFP-NSCs. (E) Nestin+ GFP protein was found in the transplantation area of GFP-NSCs group; (F) Nestin+ GFP protein was found in the transplantation area of mms6-GFP-NSCs group. [file Image_1.tif]

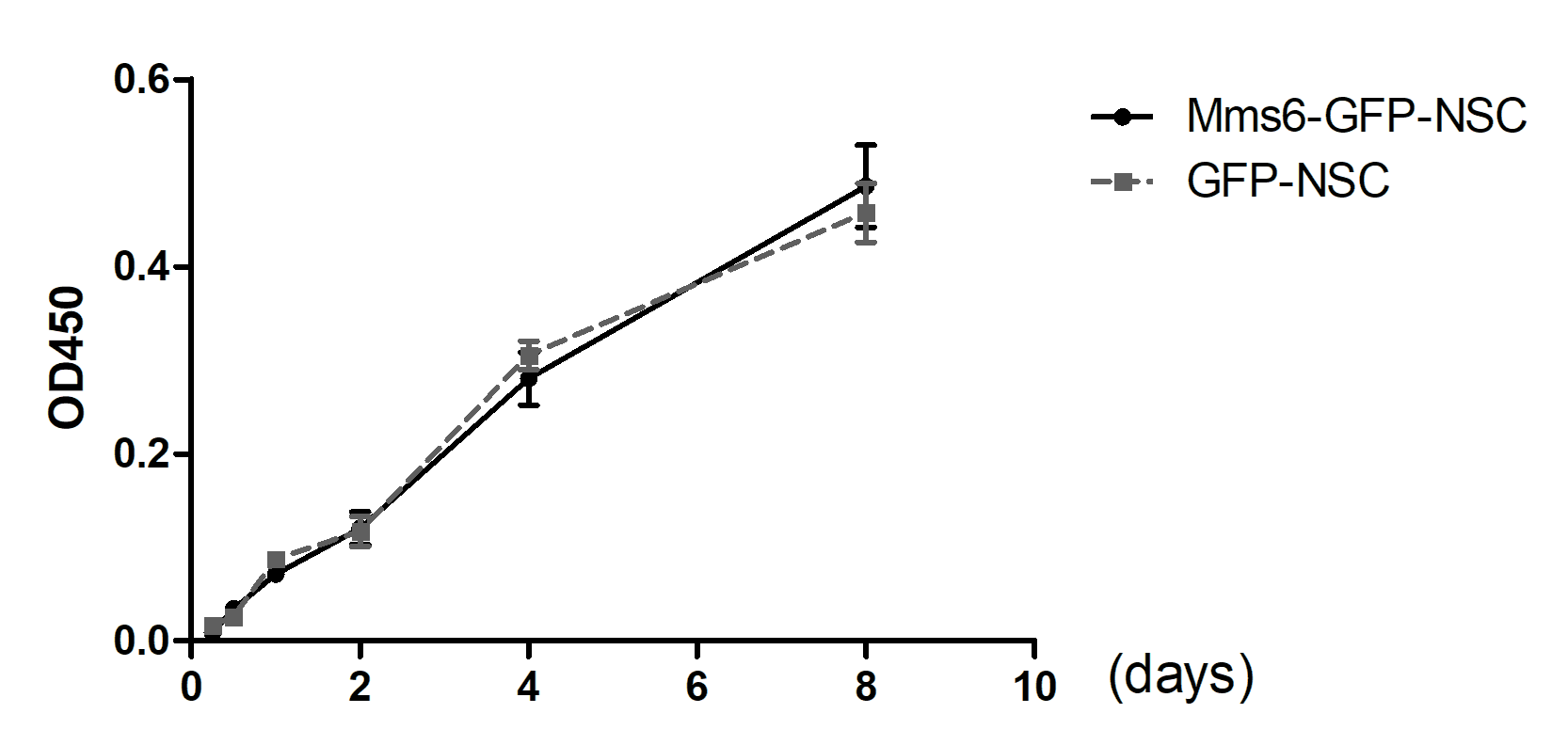

Supplement: Supplementary Figure 2 — Expression of mms6 did not affect NSCs proliferation. [file Image_2.tif]
